# Notes on Clinical Methods

**Published:** 1908-09-12

**Authors:** 


### NOTES ON CLINICAL METHODS.

### WASSERMANN'S SYPHILIS SERUM-REACTION.

Wassermann's serum-reaction for the diagnosis of syphilis is quite unlike Widal's serum-reaction for the diagnosis of typhoid fever. It does not depend upon the clumping of the spirochæte pallida. It is, on the contrary, one of an entirely different order of serum reactions, the recent discovery of which opens up a wide field of possibilities in the way of specific means of diagnostics.

The nomenclature that has arisen in connection with modern theories of immunity is very complex, and there is no possibility of defining every new term here; probably the significance of the word "complement" will be sufficiently understood to allow of the following brief explanation:—

An antigen is any substance which, injected into the living tissues, causes the latter to react and produce antibody. It has been found that antigen and its corresponding antibody have the power of fixing complement.

This may not at first sound very helpful; but to continue:—

Red blood corpuscles may be sensitised in such a way that the addition of complement to the test-tube containing them causes their solution—that is to say, causes the contents of the test-tube to become laked.

If an extract of the liver of syphilitic infants along with blood serum from a syphilitc patient is added to the test-tube containing the sensitised red corpuscles it has been found that they do not become dissolved or laked. Complement has been fixed by the combined action of syphilitic liver extract and syphilitic blood serum—antigen and antibody, and this removal of complement prevents the solution of the sensitised red corpuscles. This is the basis of Wassermann's syphilis serum-reaction.

The blood-serum of the suspected case is sent to the laboratory and there tested against the sensitised red cells, serum from a healthy individual being used as a control. According as the red cells become laked or remain unlaked the patient gives a negative or a positive Wassermann's reaction for syphilis.

The possibilities of this method of diagnosis, both in syphilis and in other specific diseases, are very obvious; so far, however, the matter is in its infancy, and many cases have yet to be tested by independent observers before its proper value can be assigned to it. It is interesting, however, to note the following results that were obtained by Fleischmann recently. He tried Wassermann's reaction in 230 cases, of which 38 were known not to be syphilitic—they were patients suffering from various ailments from exophthalmic goitre to typhoid fever; the remaining 192 cases were either known to have or to have had syphilis, or else there were strong grounds for suspecting it. Of the 38 non-syphilitic cases not one gave a positive Wassermann's reaction. Of the others, 117 or 73 per cent. gave a positive reaction, whilst in 43 or 27 per cent. the test was negative.

Without entering into a discussion of the causes of negative reactions in syphilitic cases—for instance, the possibility of past syphilis having been absolutely eradicated from the system—it seems clear that, if Fleischmann's results are confirmed, a positive reaction may be regarded as diagnostic of syphilis, although no conclusion can be drawn from a negative one.

